# *CDKN3*, *PKP2*, *ADM*, *MS4A1*, *FAM83A*, and *DKK1* Define a Posttranslational Modification (PTM)–Associated Prognostic Model for Lung Adenocarcinoma

**DOI:** 10.1155/humu/3201577

**Published:** 2026-08-24

**Authors:** Zhi Liu, Gang Li, Ling Lin

**Affiliations:** ^1^ State Key Laboratory of Advanced Medical Materials and Medical Devices, Tianjin University, Tianjin, China, tju.edu.cn; ^2^ Office of Network and Security Information, Tianjin University of Traditional Chinese Medicine, Tianjin, China, tjutcm.edu.cn; ^3^ School of Precision Instrument and Opto-Electronics Engineering, Tianjin University, Tianjin, China, tju.edu.cn; ^4^ Medical School of Tianjin University, Tianjin, China; ^5^ National Key Laboratory of Precision Measurement Technology and Instruments, Tianjin, China

**Keywords:** immune infiltration, lung adenocarcinoma, machine learning, posttranslational modification, single-cell RNA sequencing

## Abstract

**Introduction:**

Posttranslational modification (PTM) plays an important role in protein regulation and may influence tumor initiation and progression. However, the role of PTM‐related programs in lung adenocarcinoma (LUAD) remains incompletely understood.

**Materials and Methods:**

Single‐cell RNA sequencing (scRNA‐seq) data were analyzed to quantify the activity of PTM‐related gene set using the AUCell algorithm and to characterize intercellular communication using CellChat. Molecular subtypes and differentially expressed genes (DEGs) were identified using ConsensusClusterPlus and limma packages, respectively, followed by functional enrichment analysis. Univariate Cox regression, LASSO regression with 10‐fold cross‐validation, and stepwise multivariable Cox regression based on the Akaike information criterion (AIC) were used to construct a prognostic model. Immune infiltration was evaluated using ESTIMATE, CIBERSORT, MCP‐counter, and TIMER algorithms, and drug sensitivity was predicted using oncoPredict package. The IMvigor210 cohort was used for exploratory assessment of immunotherapy response. Quantitative real‐time reverse transcription PCR (qRT‐PCR) was used to measure the expressions of the model genes. CCK‐8, wound‐healing, and Transwell assays were performed to evaluate the effects of *CDKN3* knockdown on LUAD cells.

**Results:**

Comparison between two PTM groups revealed that receptor ligands such as MIF‐(CD74+CXCR4) and MIF‐(CD74+CD44) had higher communication probabilities in the high–PTM‐score group. Two molecular subtypes were identified, and a six‐gene risk model for LUAD was established based on *CDKN3*, *PKP2*, *ADM*, *MS4A1*, *FAM83A*, and *DKK1*. The high‐risk group showed a lower immune score, and drugs such as Docetaxel_1007 may have therapeutic potential for LUAD. Immunotherapy response prediction further demonstrated that the low‐risk group may be more likely to benefit from immunotherapy. In vitro experiments demonstrated that *CDKN3*, *PKP2*, *ADM*, *FAM83A*, and *DKK1* were upregulated, whereas *MS4A1* was downregulated in A549 cells compared with BEAS‐2B cells. Additionally, knockdown of *CDKN3* significantly suppressed the viability, migration, and invasion of LUAD cells.

**Conclusion:**

The PTM‐related six‐gene model showed potential for prognostic stratification and characterization of immune‐related features in LUAD. However, further prospective and experimental validation is required before direct clinical application.

## 1. Introduction

Lung cancer was the most frequently diagnosed cancer worldwide in 2022, with nearly 2.5 million new cases and 1.8 million deaths [1]. Nonsmall cell lung cancer (NSCLC) accounts for approximately 85% of all lung cancers, and lung adenocarcinoma (LUAD) is its most common histological subtype [2, 3]. Current treatment strategies for LUAD include surgical resection, radiotherapy, chemotherapy, molecularly targeted therapy, and immunotherapy, chosen based on disease stage and molecular characteristics [4]. Although immune checkpoint inhibitors (ICIs) have improved clinical outcomes in selected patients, heterogeneous responses and acquired resistance remain important challenges [5, 6]. Therefore, reliable prognostic biomarkers are needed to support patient stratification and treatment decision‐making. As posttranslational modifications (PTMs) regulate protein stability, localization, activity, and immune signaling, dysregulated PTM programs may provide a biological link between tumor molecular heterogeneity and immunotherapy response in LUAD.

PTMs are covalent modifications, often enzyme‐catalyzed, that occur after protein synthesis and expand the structural and functional diversity of the proteome [7, 8]. In LUAD, dysregulated phosphorylation, ubiquitination, glycosylation, and lactylation may reshape oncogenic signaling, protein stability, therapeutic resistance, and tumor‐immune interactions [9, 10]. For example, the PTM‐related gene *B4GALT2* has been identified as an oncogenic regulator associated with immune exclusion in LUAD [11]. However, the cell‐type‐specific activity, molecular heterogeneity, and prognostic relevance of PTM‐related programs in LUAD have not been systematically characterized by integrating single‐cell and bulk transcriptomic data.

Single‐cell RNA sequencing (scRNA‐seq) enables high‐resolution characterization of cellular heterogeneity and gene‐regulatory relationships [12, 13]. In this study, we integrated scRNA‐seq and bulk transcriptomic data to characterize PTM‐related activity and cell–cell communication, identify molecular subtypes, and construct a prognostic risk model for LUAD [14]. The novelty of this study lies in linking single‐cell PTM activity and intercellular communication with bulk‐transcriptomic molecular subtyping and prognostic modeling, followed by external‐cohort and in vitro validation.

## 2. Material and Methods

### 2.1. Data Collection

The scRNA‐seq dataset GSE189357, comprising nine lung cancer samples, was downloaded from the Gene Expression Omnibus (GEO). Bulk transcriptomic and clinical data for TCGA‐LUAD were obtained from the UCSC Xena database, and 490 primary tumor samples were retained after screening. The GSE31210 microarray dataset, comprising 226 primary LUAD samples, was downloaded from GEO and used for external validation. Specifically, TCGA‐LUAD contained 490 primary tumor samples, GSE31210 contained 226 primary LUAD samples, and GSE189357 contained nine lung cancer samples. PTM‐related genes were collected from relevant literature [15].

### 2.2. Data Preprocessing

The three datasets were preprocessed separately. For TCGA‐LUAD dataset, samples without follow‐up information were excluded, retaining only patients with an overall survival (OS) time longer than 30 days. Ensembl identifiers were converted to gene symbols. When multiple identifiers mapped to the same gene symbol, the maximum expression value was retained. For GSE31210 dataset, probes were mapped to genes according to the GPL570 platform annotation, and the maximum expression value was retained when multiple probes mapped to the same gene. For GSE189357 dataset, cells meeting the criteria of nFeature_RNA > 200, nCount_RNA < 15,000, and percent.mt < 10 were retained. The data were scaled using ScaleData [16], and principal component analysis (PCA) was performed using the RunPCA function from the Seurat R package [16]. After removing batch effects using Harmony package [17], Uniform Manifold Approximation and Projection (UMAP) was performed on the first 30 PCs. Cell clusters were identified using the FindNeighbors and FindClusters functions [16], and cell types were annotated using canonical marker genes from the CellMarker 2.0 database [18].

### 2.3. PTM Scores and Cell Communication

To systematically evaluate the potential role of PTM in LUAD, we applied the AUCell algorithm [19] to score the expression activity of PTM‐related gene sets in individual cells. AUCell algorithm was used to rank genes by expression and assess the enrichment of a given gene set within each cell, thereby quantifying PTM activity at single‐cell resolution. Based on the median AUCell score, all cells were divided into high‐ and low–PTM‐score groups. Additionally, Gene Set Enrichment Analysis (GSEA) was performed to identify differentially enriched pathways [20], and CellChat was used to compare intercellular communication [21].

### 2.4. Identification of Molecular Subtypes

Prognostic PTM‐related genes were screened by univariate Cox regression analysis. Then, a consistency matrix was constructed using ConsensusClusterPlus [22] to classify molecular subtypes of LUAD. We employed the partitioning around medoids (PAMs) algorithm with Pearson distance metric and 1000 bootstrap iterations, each sampling including 80% of the samples in training set. The number of clusters ranged from 2 to 10, and the optimal cluster number was determined by the consistency cumulative distribution function (CDF). Differentially expressed genes (DEGs) between subtypes were identified using the limma package [23] with the threshold of FDR < 0.05 and |log2 FC| > log2(2). Functional enrichment analysis of KEGG pathways and Gene Ontology (GO) biological processes was performed on these DEGs using the clusterProfiler R package [24].

### 2.5. Development of the Risk Model

Based on DEGs, further univariate Cox regression analysis was first performed. LASSO regression with 10‐fold cross‐validation (CV) was then applied using the glmnet package [25] to further select candidate genes. Subsequently, stepwise multivariate regression analysis based on the AIC was used to refine the gene set and construct the final risk model. The risk score for each patient was calculated as RiskScore = *Σ* *β* *i* × Exp(i), where *β*i represents the Cox regression coefficient of gene i and Exp(i) represents the normalized expression level of gene i. RiskScores were subsequently standardized using *z*‐score transformation and used to stratify patients into high‐ and low‐risk groups. Further, time‐dependent receiver operating characteristic (ROC) analysis was performed using the timeROC package [26] to assess the prognostic accuracy of the RiskScore, and Kaplan–Meier (KM) survival curves were generated to compare survival outcomes between the two groups.

### 2.6. Associations of the RiskScore With Immune Infiltration and Drug Sensitivities

Quantitative analysis of the immune components was conducted using the ESTIMATE algorithm [27]. Subsequently, we employed the CIBERSORT [28], MCP‐Counter [29], and TIMER algorithms [30] to estimate immune infiltration scores and assess relative abundances of immune cell types in the TCGA‐LUAD cohort.

Drug half‐maximal inhibitory concentrations (IC50 values) were predicted using oncoPredict [31]. Associations between drug sensitivity and the RiskScore were evaluated using Spearman correlation analysis, with adjusted *p* < 0.05 and |correlation coefficient| > 0.3 set as the thresholds for significance.

### 2.7. Immunotherapy Analysis in Different Risk Groups

To evaluate the predictive value of the RiskScore for immunotherapy efficacy, we first examined the correlation between the RiskScore and the expressions of immune‐related genes. The IMvigor210 cohort was selected as it provides pretreatment transcriptomic profiles, OS data, and standardized clinical‐response information for patients treated with the anti–PD‐L1 antibody atezolizumab [32]. However, this cohort comprises patients with urothelial carcinoma rather than LUAD; it was used only for exploratory cross‐cancer evaluation of the association between the RiskScore and immunotherapy response. In the IMvigor210 cohort, PD (progressive disease), complete response (CR), partial response (PR), and stable disease (SD) were employed to evaluate the clinical responses of patients to anti–PD‐L1 monoclonal antibody treatment.

### 2.8. Cell Culture and Transfection

Dulbecco′s Modified Eagle Medium (DMEM; iCell‐0001, iCell) supplemented with 10% FBS (iCell‐0500, iCell) and penicillin–streptomycin (P/S) double antibody (iCell‐15140‐122, iCell) was employed to culture human LUAD cells A549 (iCell‐h011, iCell, Shanghai, China) and human bronchial epithelial cells BEAS‐2B (C5382, BDBio, Hangzhou, China). All cells were authenticated by STR analysis and cultured in an incubator at 37°C with 5% CO_2_.

For liposome transfection, siRNA targeting *CDKN3* (cyclin‐dependent kinase inhibitor‐3) and control siRNA were purchased from GenePharma (Shanghai, China). Transfection was performed applying Lipofectamine 2000 transfection reagent (11668‐027, Invitrogen, Carlsbad, California, United States), according to the manufacturer′s instructions. The target sequence of si‐*CDKN3*#1 was 5 ^′^‐CCCAGTTCAATACAAACAAGTGA‐3 ^′^, and the target sequence of si‐*CDKN3*#2 was 5 ^′^‐ATCAGATGAAGAGCCTATTGAAG‐3 ^′^.

### 2.9. qRT‐PCR

Total RNA was extracted from BEAS‐2B and A549 cells using the TriZol Extraction Kit (15596‐026, Invitrogen, United States) following the manufacturer′s protocol. The concentration of the extracted RNA was measured, and cDNA was synthesized using a reverse transcription kit (D7178S, Beyotime, Shanghai, China). Subsequently, qRT‐PCR was performed using SYBR Green qPCR Mix (D7260, Beyotime). The amplification conditions were as follows: initial denaturation at 95°C for 30 s, followed by 45 cycles of denaturation at 94°C for 15 s, annealing at 56°C for 30 s, and extension at 72°C for 20 s [33]. Relative expression was calculated using the 2^−*ΔΔ*CT^ method, with GAPDH as the reference gene. Primer sequences are listed in Table 1.

**Table 1:** The primers utilized in this study.

Gene	Accession no.	Primers (5 ^′^–3 ^′^)	
		Forward	Reverse
*CDKN3*	NM_005192	ATGGAGGGACTCCTGACATAGC	TCTCCCAAGTCCTCCATAGCAG
*PKP2*	NM_004572	CAGGTGCTGAAGCAAACCAGAG	CCAAAGTGCTGGGATTACAGGC
*ADM*	NM_001124	GACATGAAGGGTGCCTCTCGAA	CCTGGAAGTTGTTCATGCTCTGG
*MS4A1*	NM_152866	CTGGTCCAAAACCACTCTTCAGG	GGCAATGTGGAAGAGCCCATTC
*FAM83A*	NM_032899	ATCCAGCGCCACTGTGTACTTC	CCGTGAACACATCCATCAGGATG
*DKK1*	NM_012242	GGTATTCCAGAAGAACCACCTTG	CTTGGACCAGAAGTGTCTAGCAC
*GAPDH*	NM_002046	GTCTCCTCTGACTTCAACAGCG	ACCACCCTGTTGCTGTAGCCAA

### 2.10. Cell Viability, Migration, and Invasion Assays

Transfected A549 cells were seeded into 96‐well plates at a density of 2 × 10^3^ cells per well. Cell viability was assessed at 0, 24, 48, and 72 h. At each indicated time point, 10 *μ*L of CCK‐8 solution (C0037, Beyotime) was added to each well, followed by incubation for 4 h. Absorbance at 450 nm was measured with an iMark microplate reader (Bio‐Rad, Hercules, California, United States).

Transfected A549 cells were plated into six‐well plates and cultured until a confluent monolayer formed. A scratch wound was created using a 200‐*μ*L sterile pipette tip, and the cells were then washed and incubated with serum‐free medium. After 48 h, an inverted light microscope (DP27, Olympus, Tokyo, Japan) was employed for imaging and quantification of wound closure.

Cell invasion assay was performed using 24‐well Transwell plates precoated with polycarbonate membranes (pore size: 8 *μ*m, 3422, Corning, Inc., Corning, New York, United States) and Matrigel (C0372, Beyotime). Transfected A549 cells were suspended in 200‐*μ*L serum‐free medium and seeded into the upper chamber, whereas the lower chamber contained 700‐*μ*L medium and 10% FBS. After 48 h, the cells were fixed by 4% paraformaldehyde (P0099, Beyotime) and stained by 0.1% crystal violet (C0121, Beyotime) for 30 min. Cell observation and quantification were performed from three random fields under an inverted light microscope (DP27, Olympus).

### 2.11. Statistical Analysis

The R 3.6.0 software was used for all statistical analyses. OS differences among molecular subtypes in the TCGA‐LUAD cohort were compared using the KM curve and log‐rank test. The Mann–Whitney *U* test and Kruskal–Wallis test were used for comparison between two groups and among multiple groups, respectively. The correlation between the RiskScore and immune‐related genes was evaluated by Spearman correlation analysis. For in vitro experiments, Welch′s *t*‐test was used for two‐group comparisons, whereas one‐way or two‐way ANOVA was applied for comparisons among three or more groups followed by Šídák′s multiple comparisons test. In all statistical tests, *p*.adj < 0.05 was considered as a statistical significance.

## 3. Results

### 3.1. LUAD Single‐Cell Atlas

**Figure 1:**
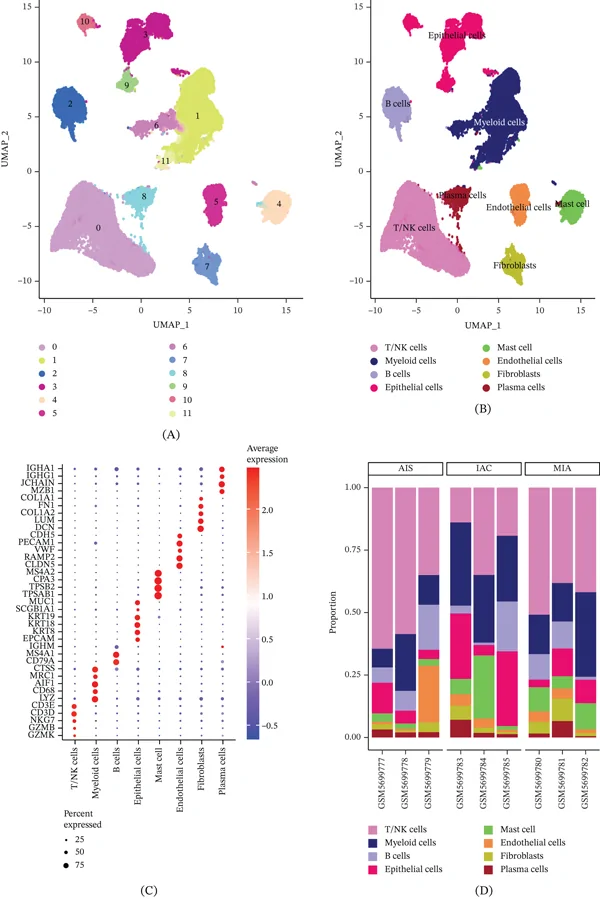
Single‐cell map of GSE189357. UMAP visualization of cell subpopulations (A) preannotation and (B) postannotation. (C) Cell subpopulations were labeled using the marker genes. (D) Proportions of each cell subpopulation in different samples.

After preprocessing, the single‐cell dataset yielded a total of 109,296 cells. Based on the clustering results, cells were grouped into 12 main cell clusters (Figure 1A,B). Each cluster was subsequently annotated using canonical marker genes, identifying eight cell types: T/NK cells, mast cells, myeloid cells, B cells, epithelial cells, endothelial cells, fibroblasts, and plasma cells (Figure 1C). Analysis of cell‐type proportions showed that T/NK cells were the most abundant (Figure 1D).

### 3.2. Analysis of PTM Score Activity and Cell Communication Differences in LUAD

**Figure 2:**
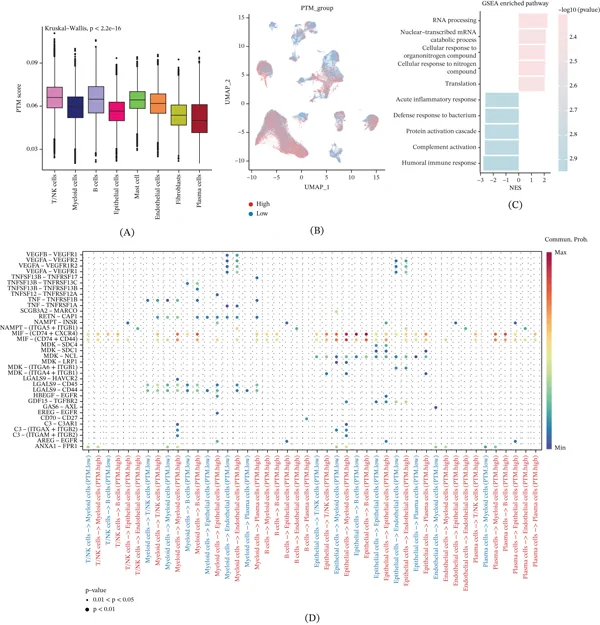
PTM activity and cell communication. (A) Scores related to PTM for each cell. (B) UMAP projection reflecting the AUCell scores of PTM in each cell, where red indicates the group with high PTM scores and blue indicates the group with low PTM scores. (C) Differences in BP enrichment between the high and low PTM score groups. (D) Bubble plot of cell communication interactions between two PTM score groups.

AUCell analysis revealed that among all cell types, NK/T cells, B cells, and endothelial cells exhibited significantly higher PTM pathway activity (*p* < 0.05, Figure 2A), suggesting that these cells may be the primary responders to PTM‐related signaling. Based on the median of the AUCell enrichment score, all cells were divided into high‐ and low–PTM‐score groups (Figure 2B). In the high–PTM‐score group, pathways such as RNA processing and cellular response to organonitrogen compounds were activated, whereas in the low–PTM‐score group, pathways such as humoral immune response and complement activation were activated (Figure 2C). It was also found that receptor‐ligand pairs such as MIF‐(CD74+CD44) and MIF‐(CD74+CXCR4) showed higher communication probabilities in the high–PTM‐score group (Figure 2D).

### 3.3. Identification of PTM‐Related Genes

**Figure 3:**
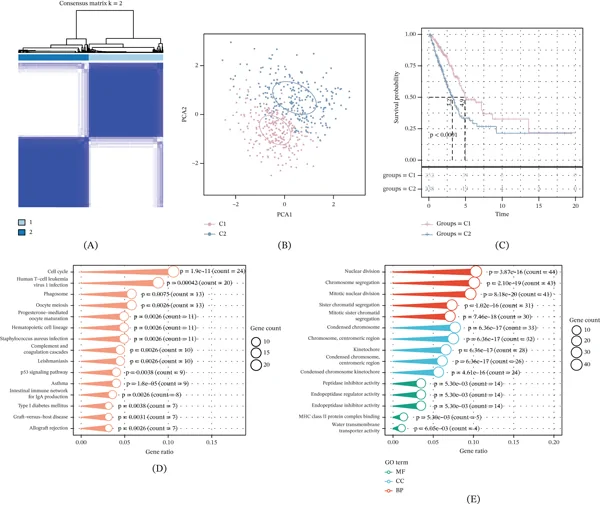
Molecular typing and enrichment analysis. (A) Consistency clustering heat map. (B) The PCA results show that there is a clear boundary between C1 and C2. (C) KM curves of OS for the two molecular subtypes. (D) DEGs between C1 and C2 subtypes were subjected to KEGG enrichment analysis. (E) GO enrichment analysis of DEGs between C1 and C2 subtypes.

Univariate Cox regression analysis identified 156 PTM‐related genes closely associated with OS (*p* < 0.05). Based on the expression profiles of these prognostic genes, consensus clustering was performed, and the optimal number of clusters was determined to be *k* = 2, resulting in two relatively stable molecular subtypes (Figure 3A). In addition, PCA results showed a clear boundary between C1 and C2, supporting the robustness of the classification (Figure 3B). Survival analysis revealed significant prognostic differences, with C1 showing more favorable prognostic outcomes and C2 associated with worse prognosis (*p* < 0.05, Figure 3C). Differential expression analysis identified 267 downregulated genes and 196 upregulated genes. Functional enrichment analysis showed that these DEGs were enriched in p53 signaling pathway, nuclear division, chromosome segregation, cell cycle, and so forth (Figure 3D,E).

### 3.4. Establishment of a Prognostic Model and Validation

**Figure 4:**
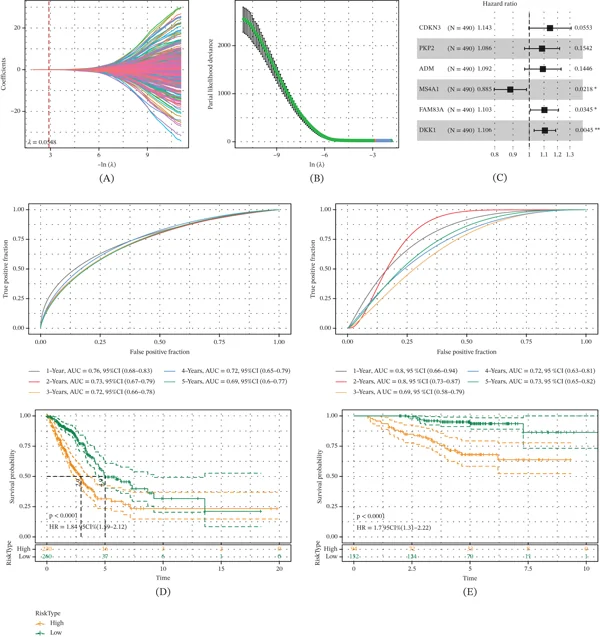
Development of the risk model and validation. (A) Trends of each independent variable across different *λ* values. (B) Confidence intervals corresponding to *λ*. (C) Forest plot of the six genes included in the prognostic model.  ^∗^ *p* < 0.05 and  ^∗∗^ *p* < 0.01. (D) The prognostic model and distribution of KM survival curve. (E) Verification of the model and KM survival curve profiling in the GSE31210 dataset.

Univariate Cox regression analysis of the subtype‐associated DEGs screened 352 prognostic genes (*p* < 0.05). LASSO regression with 10‐fold CV showed that the optimal model was obtained at *λ* = 0.0548 and retained 14 genes (Figure 4A,B). Stepwise multivariable Cox regression based on the AIC refined the model to a six‐gene RiskScore model comprising *CDKN3, PKP2* (plakophilin‐2)*, ADM* (adrenomedullin)*, MS4A1* (membrane spanning 4‐domains A1)*, FAM83A* (family with sequence similarity 83 Member A), and *DKK1* (Dickkopf‐1) (Figure 4C).

RiskScore=0.13430.08320.0880.12241∗CDKN+∗PKP+∗ADM−∗MSA  +0.098830.1011∗FAMA+∗DKK.

The standardized RiskScore classified 230 patients into the high‐risk group and 260 patients into the low‐risk group. Notably, patients in the high‐risk group had significantly shorter OS than those in the low‐risk group (*p* < 0.05; Figure 4D). Similar results were observed in the GSE31210 validation cohort (*p* < 0.05; Figure 4E).

### 3.5. Cell Experiments for Validation

**Figure 5:**
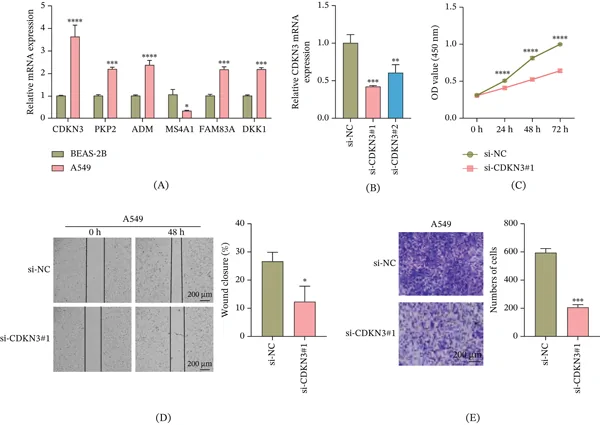
Results of cell validation. (A) Quantitative mRNA levels of *CDKN3*, *PKP2*, *ADM*, *MS4A1*, *FAM83A*, and *DKK1* in BEAS‐2B and A549 cells. (B) Validation of *CDKN3* expression after transfection. (C) CCK‐8 analysis of transfected A549 cells at 0, 24, 48, and 72 h. (D) Wound‐healing migration assay of transfected A549 cells. (E) Transwell invasion assay of transfected A549 cells. Scale bar = 200 *μ* *m*,  ^∗^ *p* < 0.05,  ^∗∗^ *p* < 0.01,  ^∗∗∗^ *p* < 0.001, and  ^∗∗∗∗^ *p* < 0.0001.

In vitro results showed that compared with BEAS‐2B cells, the expressions of *CDKN3*, *PKP2*, *ADM*, *FAM83A*, and *DKK1* were significantly upregulated, whereas *MS4A1* was significantly downregulated in A549 cells (*p* < 0.05; Figure 5A). Among the six model genes, *CDKN3* showed the greatest expression difference between A549 and BEAS‐2B cells and had the highest positive coefficient in the prognostic model. Its established role in cell‐cycle regulation was also consistent with the functional enrichment results; therefore, CDKN3 was prioritized for functional validation. The qRT‐PCR confirmed successful transfection, and si‐*CDKN3*#1, which exhibited higher knockdown efficiency, was chosen for subsequent assays (*p* < 0.05, Figure 5B). Further, functional assays showed that *CDKN3* knockdown markedly reduced the viability, migration, and invasion of LUAD cells (*p* < 0.05, Figure 5C–E).

### 3.6. Immune and Chemotherapy Drug Sensitivity

The immune score of the tumor microenvironment (TME) in the high‐risk group was markedly lower (*p* < 0.05, Figure 6A), suggesting that the suppression of immune environment may contribute to a poor prognosis. The CIBERSORT, MCP‐Counter, and TIMER analyses showed that the scores of most immune cells, especially T cells, B cells, fibroblasts, and endothelial cells, in the high‐risk group were significantly lower (*p* < 0.05, Figure 6B–D).

Figure 6Analysis of immune and candidate drugs. (A) Using ESTIMATE to analyze the immune cell infiltration in the two groups of the TCGA‐LUAD cohort. (B) CIBERSORT was applied to evaluate the disparities in immune cell scores between the two groups. (C) Using MCP‐counter to analyze the differences in immune cell scores between the two groups. (D) The TIMER algorithm was employed to assess the disparities in immune cell scores between the two groups. (E) Correlation analysis of RiskScore and key gene expression with drug IC50 values in the TCGA‐LUAD dataset. Abbreviation: ns, not significant.  ^∗^ *p* < 0.05,  ^∗∗^ *p* < 0.01, and  ^∗∗∗^ *p* < 0.001.

**Figure:**
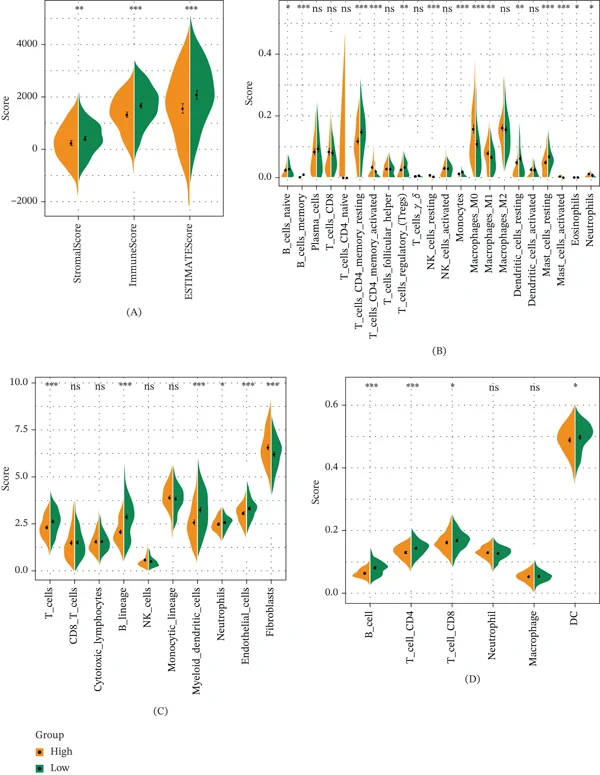
(A)

**Figure:**
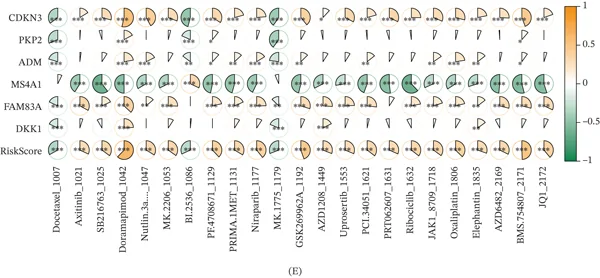
(B)

Drug sensitivity analysis revealed that the IC50 value of 23 drugs was significantly correlated with the RiskScore. Among them, Docetaxel_1007 was notably negatively correlated with all the key genes except *MS4A1* (*p* < 0.05, Figure 6E), indicating that this drug might have potential for the treatment of LUAD.

### 3.7. Correlation Analysis Between the RiskScore and Immunotherapy

**Figure 7:**
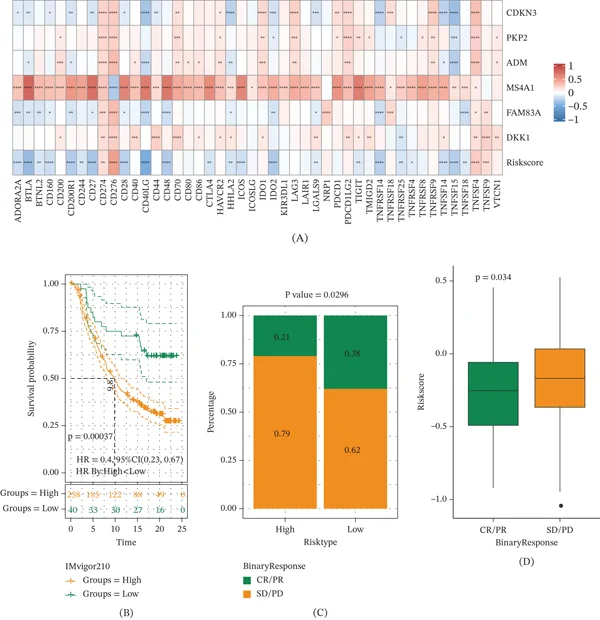
Prediction of immunotherapy in different risk groups. (A) Correlations of the RiskScore and six signature genes with immune‐related genes.  ^∗^ *p* < 0.05,  ^∗∗^ *p* < 0.01,  ^∗∗∗^ *p* < 0.001, and  ^∗∗∗∗^ *p* < 0.0001. (B) Survival differences, (C) immune response differences, and (D) differences in RiskScore among different response degrees in the IMvigor210 cohort risk groups.

Correlation analysis showed that the RiskScore and the six model genes were associated with multiple immune‐related genes, with *MS4A1* exhibiting the broadest correlations (*p* < 0.05; Figure 7A). These findings suggested that the RiskScore may reflect immune‐regulatory characteristics of the TME. Next, we analyzed the IMvigor210 cohort, which comprises patients with advanced urothelial carcinoma receiving immunotherapy. The OS of patients with high RiskScores was significantly shorter (*p* < 0.05, Figure 7B), and the proportion of patients achieving CR and PR was notably lower (*p* < 0.05, Figure 7C). Furthermore, comparison of the RiskScore distribution in different therapeutic response subtypes revealed that the RiskScore of patients with SD/PD was significantly higher than that of patients with CR and PR (*p* < 0.05, Figure 7D).

## 4. Discussion

Targeted therapy and immunotherapy have significantly improved the treatment outcomes of LUAD [34]. Among these, platinum‐based chemotherapy combined with PD‐1 axis blockade represents a key treatment strategy for lung cancer [35]. PTM, a crucial regulatory mechanism of proteins, is related to the change of protein activity, regulation of protein degradation, and tumor development [36]. This study identified six PTM‐related key genes in LUAD. It was found that the immune score of the high‐risk LUAD group was lower. The predictive analysis revealed that the low‐risk LUAD patients were more likely to benefit from immunotherapy, and that specific drugs such as Docetaxel_1007 were predicted as potential treatments for LUAD.

This study identified six PTM‐related key genes for LUAD, including *CDKN3*, *PKP2*, *ADM*, *MS4A1*, *FAM83A*, and *DKK1*. *CDKN3* can dephosphorylate serine/threonine residues, mainly influencing tumors by regulating the cell cycle and mitosis [37]. Consistently, the DEGs identified were mainly enriched in the cell cycle and p53 signaling pathway. Past research showed that *CDKN3* contributes to the treatment of LUAD [38]. *PKP2*, a constituent of the desmosome complex, is widely recognized for its role in cell–cell adhesion [39]. Previous studies have found that targeting the miR‐7‐5p/*PKP2* pathway can overcome the potential of NSCLC radiotherapy resistance [40], and that *PKP2* is a key driver of lung cancer radioresistance [41]. *ADM* is a vasodilator peptide closely related to functions such as vasodilation, angiogenesis, and anti‐inflammation [42]. In patients with LUAD, *ADM* has the potential to act as a predictive marker for cardiac ischemia [43]. *MS4A1*, which is primarily distributed in B‐cell clusters, plays a pivotal role in B‐cell differentiation [44]. *MS4A1* regulates the infiltration of M1‐polarized tumor‐associated macrophages, angiogenesis and cancer progression in LUAD through the HIPPO pathway [45]. *FAM83A* directly interacts with *β*‐catenin in pancreatic cancer and affects the phosphorylation and degradation process in PTM [46]. By regulating the Wnt and Hippo signaling pathways and the EMT program, it promotes the proliferation and invasion of lung cancer cells [47]. Moreover, *FAM83A* can promote the expression of erythroblastic leukemia viral oncogene homolog 2 (*ErbB2*) in PTM through the E3 ubiquitin ligase *STUB1* [48]. *DKK1* is a key component of the Wnt/*β*‐catenin signaling pathway and acts as a canonical Wnt antagonist [49]. *DKK1* promotes tumor immune evasion and inhibits anti–PD‐1 therapy by inducing immunosuppressive macrophages in gastric cancer [50]. Collectively, these six genes affect the cell cycle or other related pathways through PTM, thereby influencing the occurrence of the cancer.

The high‐risk group exhibited lower infiltration scores for fibroblasts, B cells, endothelial cells, and T cells. Fibroblastic reticular cells promote the formation of a protective intratumoral T‐cell niche in lung cancer, and T‐cell activity is indispensable for mounting effective antitumor immune responses [51]. Chimeric antigen receptor T‐cell (CAR‐T cell) immunotherapy is a novel immunotherapy approach that has been extensively studied in LUAD [52]. The spatial structure of T cells is closely related to immune evasion in LUAD [53]. In LUAD, B cells play a dual role in antitumor immunity and protumor immunity, and the balance among different B‐cell subtypes determines their net impact on tumor immunity [54]. *ADM* enhances the barrier function of human pulmonary endothelial cell monolayers [55]. *DKK1* is related to the drug resistance of some drugs in LUAD and can drive tumor progression [56]. Collectively, the above evidence suggests that the related key genes function through these cells in LUAD. In the IMvigor210 cohort, the RiskScore was associated with survival and immunotherapy‐response categories, indicating that it may capture immune‐related biological features shared across tumor types. However, as IMvigor210 comprises patients with urothelial carcinoma rather than LUAD, these findings represent exploratory cross‐cancer evidence and do not establish LUAD‐specific predictive utility. Validation in independent LUAD cohorts receiving ICIs is required. Docetaxel_1007 was predicted as a potential drug for the treatment of LUAD. Docetaxel, a taxane antitumor drug, exerts its effect through microtubule polymerization and induction of cell cycle arrest [57]. It has been confirmed as one of the important therapeutic drugs for LUAD [58]. Therefore, PTM‐based personalized treatment may hold clinical value for LUAD.

Several limitations should be acknowledged. First, the prognostic model was retrospectively developed using public transcriptomic datasets, necessitating validation in larger, prospective, multicenter LUAD cohorts. Second, functional validation was limited to *CDKN3* knockdown in a single LUAD cell line, and the biological functions and PTM‐related mechanisms of the remaining model genes should be investigated using additional LUAD cell lines, animal models, and clinical specimens. Third, drug sensitivity was computationally predicted, and immunotherapy performance was explored using a non‐LUAD cohort. Therefore, pharmacological experiments and independent LUAD cohorts with patients receiving ICIs are required before clinical translation of the six genes.

## 5. Conclusion

This study established a six‐gene PTM‐related prognostic model for LUAD by integrating single‐cell and bulk transcriptomic analyses. The model was associated with patient survival, immune microenvironment characteristics, predicted drug sensitivity, and exploratory immunotherapy‐response patterns. These findings provide a framework for further investigation of PTM‐related heterogeneity and patient stratification in LUAD; however, prospective and mechanistic validation remains necessary.

**Nomenclature**
ADM: adrenomedullin
AIC: Akaike information criterion
ANOVA: analysis of variance
AUC: area under the curve
CDKN3: cyclin‐dependent kinase inhibitor‐3
CR: complete response
CV: cross‐validation
DEG: differentially expressed gene
DKK1: Dickkopf‐1
DMEM: Dulbecco′s Modified Eagle Medium
EMT: epithelial‐mesenchymal transition
ErbB2: erythroblastic leukemia viral oncogene homolog 2
FAM83A: family with sequence similarity 83 member A
FBS: fetal bovine serum
FC: fold change
FDR: false discovery rate
GEO: Gene Expression Omnibus
GO‐BP: Gene Ontology‐Biological Process
GSEA: Gene Set Enrichment Analysis
IC50: half‐maximal inhibitory concentration
KEGG: Kyoto Encyclopedia of Genes and Genomes
KM: Kaplan–Meier
LASSO: least absolute shrinkage and selection operator
LUAD: lung adenocarcinoma
MHC: major histocompatibility complex
MS4A1: membrane spanning 4‐domains A1
NK: natural killer
NSCLC: nonsmall cell lung cancer
OS: overall survival
P/S: penicillin–streptomycin
PCA: principal component analysis
PD: progressive disease
PD‐1: programmed death 1
PD‐L1: programmed death‐ligand 1
PKP2: plakophilin‐2
PR: partial response
PTM: posttranslational modification
qRT‐PCR: quantitative real‐time reverse transcription PCR
SCLC: small cell lung cancer
scRNA‐seq: single‐cell RNA sequencing
SD: stable disease
STR: short tandem repeat
TCGA: The Cancer Genome Atlas
UCSC: University of California, Santa Cruz
UMAP: Uniform Manifold Approximation and Projection
Wnt: wingless‐related integration site

## Author Contributions

All authors contributed to this present work. Zhi Liu designed the study and interpreted the data. Gang Li acquired the data. Ling Lin drafted and revised the manuscript.

## Funding

This work was supported by the National Natural Science Foundation of China, 10.13039/501100001809, 881503456.

## Disclosure

All authors have read and approved the manuscript.

## Ethics Statement

Ethical approval was not required for this study because it did not involve any human or animal experiments.

## Consent

The authors have nothing to report.

## Conflicts of Interest

The authors declare no conflicts of interest.

## Data Availability

The datasets generated and/or analyzed during the current study are available in the [GSE189357] repository (https://www.ncbi.nlm.nih.gov/geo/query/acc.cgi?acc=GSE189357) and [GSE31210] repository (https://www.ncbi.nlm.nih.gov/geo/query/acc.cgi?acc=GSE31210).

## References

[1] Bray F. , Laversanne M. , Sung H. , Ferlay J. , Siegel R. L. , Soerjomataram I. , and Jemal A. , Global Cancer Statistics 2022: GLOBOCAN Estimates of Incidence and Mortality Worldwide for 36 Cancers in 185 Countries, CA: A Cancer Journal for Clinicians. (2024) 74, no. 3, 229–263, 10.3322/caac.21834, 38572751. 38572751

[2] Herbst R. S. , Morgensztern D. , and Boshoff C. , The Biology and Management of Non-Small Cell Lung Cancer, Nature. (2018) 553, no. 7689, 446–454, 10.1038/nature25183. 29364287

[3] Wang C. , Ding S. , Sun B. , Shen L. , Han Z. , and Huang H. , Hsa-miR-4271 Downregulates the Expression of Constitutive Androstane Receptor and Enhances in Vivo the Sensitivity of Non-Small Cell Lung Cancer to Gefitinib, Pharmacological Research. (2020) 161, 105110, 10.1016/j.phrs.2020.105110, 32755614. 32755614

[4] Hui J. J. , Ding S. J. , Qin B. D. , and Ding N. , Case report: Aumolertinib Plus Gumarontinib in a Patient With EGFR Mutated Non-Small-Cell Lung Cancer Harboring Acquired MET Amplification Following Progression on Afatinib Plus Crizotinib, Frontiers in Pharmacology. (2025) 16, 1525251, 10.3389/fphar.2025.1525251, 40028158. 40028158 PMC11868761

[5] Lu X. , Ma L. , Yin X. , Ji H. , Qian Y. , Zhong S. , Yan A. , and Zhang Y. , The Impact of Tobacco Exposure on Tumor Microenvironment and Prognosis in Lung Adenocarcinoma by Integrative Analysis of Multi-Omics Data, International Immunopharmacology. (2021) 101, pt. B, 108253, 10.1016/j.intimp.2021.108253, 34700112. 34700112

[6] Shi L. , Du W. , Miao J. , Peng X. Y. , Li W. , Ur-Rehman A. , Ge M. W. , Shen L. T. , Feng R. , Zhong K. , and Gao S. Q. , Immunotherapy Checkpoint Inhibitor-Combination Therapy Versus Chemotherapy Alone Among Chinese Population in Cholangiocarcinoma Treatment: A Systematic Review and Meta-Analysis, European Journal of Clinical Pharmacology. (2026) 82, no. 2, 10.1007/s00228-025-03948-x, 41546709. 41546709

[7] Shroff G. S. , Strange C. D. , Altan M. , Carter B. W. , Ahuja J. , Godoy M. C. B. , Truong M. T. , and Vlahos I. , Post-Immunotherapy Imaging in Lung Cancer, Clinical Radiology. (2022) 77, no. 1, 44–57, 10.1016/j.crad.2021.05.003, 34103147. 34103147

[8] Sun C. , Guan H. , Li J. , and Gu Y. , RBM15-Mediated m6A Modification of XPR1 Promotes the Malignant Progression of Lung Adenocarcinoma, Naunyn-Schmiedeberg′s Archives of Pharmacology. (2025) 398, no. 7, 9279–9290, 10.1007/s00210-025-03849-x, 39928150. 39928150

[9] Iozzo M. , Pardella E. , Giannoni E. , and Chiarugi P. , The Role of Protein Lactylation: A Kaleidoscopic Post-Translational Modification in Cancer, Molecular Cell. (2025) 85, no. 7, 1263–1279, 10.1016/j.molcel.2025.02.011, 40073861. 40073861

[10] Sun Y. , Kong W. , Zhu X. , Chu X. , Wang X. , Zhu G. , He X. , and Li J. , Novel Insights in the Diagnosis and Treatment of Non-Small Cell Lung Cancer: Post-Translational Modification of Proteins, Biochimica et Biophysica Acta Reviews on Cancer. (2026) 1881, no. 1, 189507, 10.1016/j.bbcan.2025.189507, 41354328. 41354328

[11] Zhang P. , Wang D. , Zhou G. , Jiang S. , Zhang G. , Zhang L. , and Zhang Z. , Novel Post-Translational Modification Learning Signature Reveals B4GALT2 as an Immune Exclusion Regulator in Lung Adenocarcinoma, Journal for Immunotherapy of Cancer. (2025) 13, no. 2, e010787, 10.1136/jitc-2024-010787, 40010763. 40010763 PMC11865799

[12] Ye L. , Tang X. , Zhong J. , Li W. , Xu T. , Xiang C. , Gu J. , Feng H. , Luo Q. , and Wang G. , Unraveling the Complex Pathophysiology of White Matter Hemorrhage in Intracerebral Stroke: A Single-Cell RNA Sequencing Approach, CNS Neuroscience & Therapeutics. (2024) 30, no. 3, e14652, 10.1111/cns.14652, 38433011. 38433011 PMC10909628

[13] Yu Y. , Liu M. , Wang Z. , Liu Y. , Yao M. , Wang L. , and Zhong L. , Identification of Oxidative Stress Signatures of Lung Adenocarcinoma and Prediction of Patient Prognosis or Treatment Response With Single-Cell RNA Sequencing and Bulk RNA Sequencing Data, International Immunopharmacology. (2024) 137, 112495, 10.1016/j.intimp.2024.112495. 38901238

[14] Chen D. , Xu L. , Xing H. , Shen W. , Song Z. , Li H. , Zhu X. , Li X. , Wu L. , Jiao H. , Li S. , Yan J. , He Y. , and Yan D. , Sangerbox 2: Enhanced Functionalities and Update for a Comprehensive Clinical Bioinformatics Data Analysis Platform, iMeta. (2024) 3, no. 5, e238, 10.1002/imt2.238, 39429873. 39429873 PMC11487553

[15] Chen S. , Li Y. , Hu J. , Li H. , Hu C. , Zhao J. , Qian H. , Bai S. , Tang Z. , and Feng Y. , Integrating Bulk RNA-seq, scRNA-seq, and Spatial Transcriptomics Data to Identify Novel Post-Translational Modification-Related Molecular Subtypes and Therapeutic Responses in Hepatocellular Carcinoma, Cancer Cell International. (2025) 25, no. 1, 10.1186/s12935-025-03964-y, 41044669. PMC12495838 41044669

[16] Tan Z. , Chen X. , Zuo J. , Fu S. , Wang H. , and Wang J. , Comprehensive Analysis of scRNA-Seq and Bulk RNA-Seq Reveals Dynamic Changes in the Tumor Immune Microenvironment of Bladder Cancer and Establishes a Prognostic Model, Journal of Translational Medicine. (2023) 21, no. 1, 10.1186/s12967-023-04056-z, 36973787. PMC10044739 36973787

[17] Cao G. , Xuan X. , Li Y. , Hu J. , Zhang R. , Jin H. , and Dong H. , Single-cell RNA Sequencing Reveals the Vascular Smooth Muscle Cell Phenotypic Landscape in Aortic Aneurysm, Cell Communication and Signaling: CCS. (2023) 21, no. 1, 10.1186/s12964-023-01120-5, 37189183. PMC10184349 37189183

[18] Luo H. , Xia X. , Huang L. B. , An H. , Cao M. , Kim G. D. , Chen H. N. , Zhang W. H. , Shu Y. , Kong X. , Ren Z. , Li P. H. , Liu Y. , Tang H. , Sun R. , Li C. , Bai B. , Jia W. , Liu Y. , Zhang W. , Yang L. , Peng Y. , Dai L. , Hu H. , Jiang Y. , Hu Y. , Zhu J. , Jiang H. , Li Z. , Caulin C. , Park J. , and Xu H. , Pan-Cancer Single-Cell Analysis Reveals the Heterogeneity and Plasticity of Cancer-Associated Fibroblasts in the Tumor Microenvironment, Nature Communications. (2022) 13, no. 1, 10.1038/s41467-022-34395-2, 36333338. PMC9636408 36333338

[19] Huang H. , Chen K. , Zhu Y. , Hu Z. , Wang Y. , Chen J. , Li Y. , Li D. , and Wei P. , A Multi-Dimensional Approach to Unravel the Intricacies of Lactylation Related Signature for Prognostic and Therapeutic Insight in Colorectal Cancer, Journal of Translational Medicine. (2024) 22, no. 1, 10.1186/s12967-024-04955-9, 38419085. PMC10900655 38419085

[20] Subramanian A. , Tamayo P. , Mootha V. K. , Mukherjee S. , Ebert B. L. , Gillette M. A. , Paulovich A. , Pomeroy S. L. , Golub T. R. , Lander E. S. , and Mesirov J. P. , Gene Set Enrichment Analysis: A Knowledge-Based Approach for Interpreting Genome-Wide Expression Profiles, Proceedings of the National Academy of Sciences of the United States of America. (2005) 102, no. 43, 15545–15550, 10.1073/pnas.0506580102, 16199517. 16199517 PMC1239896

[21] Jin S. , Guerrero-Juarez C. F. , Zhang L. , Chang I. , Ramos R. , Kuan C. H. , Myung P. , Plikus M. V. , and Nie Q. , Inference and Analysis of Cell-Cell Communication Using CellChat, Nature Communications. (2021) 12, no. 1, 10.1038/s41467-021-21246-9, 33597522. PMC7889871 33597522

[22] Wilkerson M. D. and Hayes D. N. , ConsensusClusterPlus: A Class Discovery Tool With Confidence Assessments and Item Tracking, Bioinformatics. (2010) 26, no. 12, 1572–1573, 10.1093/bioinformatics/btq170, 20427518. 20427518 PMC2881355

[23] Qiu J. , Zhou T. , Wang D. , Hong W. , Qian D. , Meng X. , and Liu X. , Pan-Cancer Analysis Identifies AIMP2 as a Potential Biomarker for Breast Cancer, Current Genomics. (2023) 24, no. 5, 307–329, 10.2174/0113892029255941231014142050, 38235352. 38235352 PMC10790333

[24] Wu T. , Hu E. , Xu S. , Chen M. , Guo P. , Dai Z. , Feng T. , Zhou L. , Tang W. , Zhan L. , Fu X. , Liu S. , Bo X. , and Yu G. , Cluster Profiler 4.0: A Universal Enrichment Tool for Interpreting Omics Data, Innovation. (2021) 2, no. 3, 100141, 10.1016/j.xinn.2021.100141, 34557778. 34557778 PMC8454663

[25] Yang L. , Qu Q. , Hao Z. , Sha K. , Li Z. , and Li S. , Powerful Identification of Large Quantitative Trait Loci Using Genome-Wide R/glmnet-Based Regression, Journal of Heredity. (2022) 113, no. 4, 472–478, 10.1093/jhered/esac006, 35134967. 35134967

[26] Li X. , Zhang L. , Liu C. , He Y. , Li X. , Xu Y. , Gu C. , Wang X. , Wang S. , Zhang J. , and Liu J. , Construction of Mitochondrial Quality Regulation Genes-Related Prognostic Model Based on Bulk-RNA-seq Analysis in Multiple Myeloma, Biofactors. (2025) 51, no. 1, e2135, 10.1002/biof.2135, 39446019. 39446019

[27] Yoshihara K. , Shahmoradgoli M. , Martínez E. , Vegesna R. , and Kim H. , Inferring Tumour Purity and Stromal and Immune Cell Admixture From Expression Data, Nature Communications. (2013) 4, no. 1, 10.1038/ncomms3612, 24113773. PMC3826632 24113773

[28] Chen B. , Khodadoust M. S. , Liu C. L. , Newman A. M. , and Alizadeh A. A. , Profiling Tumor Infiltrating Immune Cells With CIBERSORT, Methods in Molecular Biology. (2018) 1711, 243–259, 10.1007/978-1-4939-7493-1_12, 29344893. 29344893 PMC5895181

[29] Shu J. , Jiang J. , and Zhao G. , Identification of Novel Gene Signature for Lung Adenocarcinoma by Machine Learning to Predict Immunotherapy and Prognosis, Frontiers in Immunology. (2023) 14, 1177847, 10.3389/fimmu.2023.1177847, 37583701. 37583701 PMC10424935

[30] Li T. , Fu J. , Zeng Z. , Cohen D. , Li J. , Chen Q. , Li B. , and Liu X. S. , TIMER2.0 for Analysis of Tumor-Infiltrating Immune Cells, Nucleic Acids Research. (2020) 48, no. W1, W509–W514, 10.1093/nar/gkaa407, 32442275. 32442275 PMC7319575

[31] Maeser D. , Gruener R. F. , and Huang R. S. , OncoPredict: An R Package for predictingin vivoor Cancer Patient Drug Response and Biomarkers From Cell Line Screening Data, Briefings in Bioinformatics. (2021) 22, no. 6, bbab260, 10.1093/bib/bbab260, 34260682. 34260682 PMC8574972

[32] Rosenberg J. E. , Hoffman-Censits J. , Powles T. , van der Heijden M. S. , Balar A. V. , Necchi A. , Dawson N. , O′Donnell P. H. , Balmanoukian A. , Loriot Y. , Srinivas S. , Retz M. M. , Grivas P. , Joseph R. W. , Galsky M. D. , Fleming M. T. , Petrylak D. P. , Perez-Gracia J. L. , Burris H. A. , Castellano D. , Canil C. , Bellmunt J. , Bajorin D. , Nickles D. , Bourgon R. , Frampton G. M. , Cui N. , Mariathasan S. , Abidoye O. , Fine G. D. , and Dreicer R. , Atezolizumab in Patients With Locally Advanced and Metastatic Urothelial Carcinoma Who Have Progressed Following Treatment With Platinum-Based Chemotherapy: A Single-Arm, Multicentre, Phase 2 Trial, Lancet. (2016) 387, no. 10031, 1909–1920, 10.1016/s0140-6736(16)00561-4, 26952546. 26952546 PMC5480242

[33] Bustin S. A. , Benes V. , Garson J. A. , Hellemans J. , Huggett J. , Kubista M. , Mueller R. , Nolan T. , Pfaffl M. W. , Shipley G. L. , Vandesompele J. , and Wittwer C. T. , The MIQE Guidelines: Minimum Information for Publication of Quantitative Real-Time PCR Experiments, Clinical Chemistry. (2009) 55, no. 4, 611–622, 10.1373/clinchem.2008.112797. 19246619

[34] Meyer M. L. , Fitzgerald B. G. , Paz-Ares L. , Cappuzzo F. , Jänne P. A. , Peters S. , and Hirsch F. R. , New Promises and Challenges in the Treatment of Advanced Non-Small-Cell Lung Cancer, Lancet. (2024) 404, no. 10454, 803–822, 10.1016/s0140-6736(24)01029-8. 39121882

[35] Zugazagoitia J. , Osma H. , Baena J. , Ucero A. C. , and Paz-Ares L. , Facts and Hopes on Cancer Immunotherapy for Small Cell Lung Cancer, Clinical cancer research: an official journal of the American Association for Cancer Research. (2024) 30, no. 14, 2872–2883, 10.1158/1078-0432.Ccr-23-1159, 38630789. 38630789

[36] Park M. K. , Lee H. , and Lee C. H. , Post-Translational Modification of ZEB Family Members in Cancer Progression, International Journal of Molecular Sciences. (2022) 23, no. 23, 15127, 10.3390/ijms232315127, 36499447. 36499447 PMC9737314

[37] Zhang C. , Shen Q. , Gao M. , Li J. , and Pang B. , The role of Cyclin Dependent Kinase Inhibitor 3 (CDKN3) in promoting human tumors: Literature Review and Pan-Cancer Analysis, Heliyon. (2024) 10, no. 4, e26061, 10.1016/j.heliyon.2024.e26061, 38380029. 38380029 PMC10877342

[38] Luan Y. , Liang C. , Han Q. , Zhou X. , Yang N. , and Zhao L. , The Systematic Analysis of Genes Related to Butyrate Metabolism Suggests That CDKN3 Could Serve as a Promising Therapeutic Target for Lung Adenocarcinoma Treatment, BMC Cancer. (2025) 25, no. 1, 10.1186/s12885-024-13409-w, 39806313. PMC11726977 39806313

[39] Novelli V. , Malkani K. , and Cerrone M. , Pleiotropic Phenotypes Associated With PKP2 Variants, Frontiers in Cardiovascular Medicine. (2018) 5, 10.3389/fcvm.2018.00184, 30619891. PMC6305316 30619891

[40] Zhang F. , Yang J. , Qiu X. L. , He J. , Cheng C. , and Sang Y. , Low Expression of miR-7-5p Promotes Resistance to Radiotherapy in Lung Cancer Through Direct Upregulation of PKP2 Expression, Scientific Reports. (2025) 15, no. 1, 10.1038/s41598-025-01001-6, 40374728. PMC12081904 40374728

[41] Cheng C. , Pei X. , Li S. W. , Yang J. , Li C. , Tang J. , Hu K. , Huang G. , Min W. P. , and Sang Y. , CRISPR/Cas 9 Library Screening Uncovered Methylated PKP2 as a Critical Driver of Lung Cancer Radioresistance by Stabilizing *β*-Catenin, Oncogene. (2021) 40, no. 16, 2842–2857, 10.1038/s41388-021-01692-x, 33742119. 33742119

[42] Martínez-Herrero S. and Martínez A. , Adrenomedullin: Not Just Another Gastrointestinal Peptide, Biomolecules. (2022) 12, no. 2, 10.3390/biom12020156, 35204657. PMC8961556 35204657

[43] Wu C. , Lin D. W. , Jiang Y. W. , Jiang F. , Wang Z. X. , and Wang Y. S. , Relationship Between Serum Concentration of Adrenomedullin and Myocardial Ischemic T Wave Changes in Patients With Lung Cancer, Frontiers in Cardiovascular Medicine. (2022) 9, 836993, 10.3389/fcvm.2022.836993, 35355972. 35355972 PMC8959127

[44] Gao M. , Wang M. , Zhou S. , Hou J. , He W. , Shu Y. , and Wang X. , Machine Learning-Based Prognostic Model of Lactylation-Related Genes for Predicting Prognosis and Immune Infiltration in Patients With Lung Adenocarcinoma, Cancer Cell International. (2024) 24, no. 1, 10.1186/s12935-024-03592-y, 39696439. PMC11656871 39696439

[45] Gao W. , Wang R. , Yang S. , Shi Y. , Cui M. , Jiang R. , Li R. , Yu Y. , Jia D. , and Che D. , MS4A1 Regulates M1-Polarized Tumor-Associated Macrophage Infiltration, Angiogenesis, and Cancer Progression Through the HIPPO Pathway in Lung Adenocarcinoma, Cancer Immunology, Immunotherapy: CII. (2025) 74, no. 12, 10.1007/s00262-025-04213-x, 41182411. PMC12583355 41182411

[46] Zhou C. , Zhu X. , Liu N. , Dong X. , Zhang X. , Huang H. , Tang Y. , Liu S. , Hu M. , Wang M. , Deng X. , Li S. , Zhang R. , Huang Y. , Lyu H. , Xiao S. , Luo S. , Ali D. W. , Michalak M. , Chen X. Z. , Wang Z. , and Tang J. , B-Lymphoid Tyrosine Kinase-Mediated FAM83A Phosphorylation Elevates Pancreatic Tumorigenesis Through Interacting With *β*-Catenin, Signal Transduction and Targeted Therapy. (2023) 8, no. 1, 10.1038/s41392-022-01268-5, 36797256. PMC9935901 36797256

[47] Zheng Y. W. , Li Z. H. , Lei L. , Liu C. C. , Wang Z. , Fei L. R. , Yang M. Q. , Huang W. J. , and Xu H. T. , FAM83A Promotes Lung Cancer Progression by Regulating the Wnt and Hippo Signaling Pathways and Indicates Poor Prognosis, Frontiers in Oncology. (2020) 10, 10.3389/fonc.2020.00180, 32195172. PMC7066079 32195172

[48] Yuan Y. , Hao L. , Huang J. S. , Zhao F. Y. , Ju Y. H. , Wang J. M. , Zhang T. , Li B. Q. , and Yu Z. W. , Promotion of Stem Cell-Like Phenotype of Lung Adenocarcinoma by FAM83A via Stabilization of ErbB2, Cell Death & Disease. (2024) 15, no. 6, 10.1038/s41419-024-06853-w, 38942760. PMC11213963 38942760

[49] Jiang H. , Zhang Z. , Yu Y. , Chu H. Y. , Yu S. , Yao S. , Zhang G. , and Zhang B. T. , Drug Discovery of DKK1 Inhibitors, Frontiers in Pharmacology. (2022) 13, 847387, 10.3389/fphar.2022.847387, 35355709. 35355709 PMC8959454

[50] Shi T. , Zhang Y. , Wang Y. , Song X. , Wang H. , Zhou X. , Liang K. , Luo Y. , Che K. , Wang X. , Pan Y. , Liu F. , Yang J. , Liu Q. , Yu L. , Liu B. , and Wei J. , DKK1 Promotes Tumor Immune Evasion and Impedes Anti-PD-1 Treatment by Inducing Immunosuppressive Macrophages in Gastric Cancer, Cancer Immunology Research. (2022) 10, no. 12, 1506–1524, 10.1158/2326-6066.Cir-22-0218, 36206576. 36206576

[51] Onder L. , Papadopoulou C. , Lütge A. , Cheng H. W. , Lütge M. , Perez-Shibayama C. , Gil-Cruz C. , De Martin A. , Kurz L. , Cadosch N. , and Pikor N. B. , Fibroblastic Reticular Cells Generate Protective Intratumoral T Cell Environments in Lung Cancer, Cell. (2025) 188, no. 2, 430–446, 10.1016/j.cell.2024.10.042, 39566495. 39566495

[52] Jaspers J. E. , Khan J. F. , Godfrey W. D. , Lopez A. V. , Ciampricotti M. , Rudin C. M. , and Brentjens R. J. , IL-18-Secreting CAR T Cells Targeting DLL3 Are Highly Effective in Small Cell Lung Cancer Models, Journal of Clinical Investigation. (2023) 133, no. 9, 10.1172/jci166028, 36951942. PMC10145930 36951942

[53] Enfield K. S. S. , Colliver E. , Lee C. , Magness A. , Moore D. A. , Sivakumar M. , Grigoriadis K. , Pich O. , Karasaki T. , Hobson P. S. , Levi D. , Veeriah S. , Puttick C. , Nye E. L. , Green M. , Dijkstra K. K. , Shimato M. , Akarca A. U. , Marafioti T. , Salgado R. , Hackshaw A. , TRACERx consortium , Jamal-Hanjani M. , van Maldegem F. , McGranahan N. , Glass B. , Pulaski H. , Walk E. , Reading J. L. , Quezada S. A. , Hiley C. T. , Downward J. , Sahai E. , Swanton C. , and Angelova M. , Spatial Architecture of Myeloid and T Cells Orchestrates Immune Evasion and Clinical Outcome in Lung Cancer, Cancer Discovery. (2024) 14, no. 6, 1018–1047, 10.1158/2159-8290.Cd-23-1380, 38581685. 38581685 PMC11145179

[54] Leong T. L. and Bryant V. L. , B Cells in Lung Cancer-Not Just a Bystander Cell: A Literature Review, Translational Lung Cancer Research. (2021) 10, no. 6, 2830–2841, 10.21037/tlcr-20-788. 34295681 PMC8264333

[55] Yoon Y. , Duffy S. R. , Kirk S. E. , Promnares K. , Karki P. , Birukova A. A. , Birukov K. G. , and Yuan Y. , Adrenomedullin-RAMP2 Enhances Lung Endothelial Cell Homeostasis Under Shear Stress, Cells. (2026) 15, no. 2, 10.3390/cells15020152, 41597226. PMC12839701 41597226

[56] Choi M. , Choi Y. J. , Lee Y. J. , Lee Y. , Chung J. H. , and Kang K. W. , Dickkopf-1 Promotes Tumor Progression of Gefitinib- Resistant Non-Small Cell Lung Cancer Through Cancer Cell-Fibroblast Interactions, Experimental Hematology & Oncology. (2025) 14, no. 1, 10.1186/s40164-025-00616-9, 40025612. PMC11871833 40025612

[57] Gupta R. , Kadhim M. M. , Turki Jalil A. , Qasim Alasheqi M. , Alsaikhan F. , Khalimovna Mukhamedova N. , Alexis Ramírez-Coronel A. , Hassan Jawhar Z. , Ramaiah P. , and Najafi M. , The Interactions of Docetaxel With Tumor Microenvironment, International Immunopharmacology. (2023) 119, 110214, 10.1016/j.intimp.2023.110214. 37126985

[58] Ahn M. J. , Tanaka K. , Paz-Ares L. , Cornelissen R. , Girard N. , Pons-Tostivint E. , Vicente Baz D. , Sugawara S. , Cobo M. , Pérol M. , Mascaux C. , Poddubskaya E. , Kitazono S. , Hayashi H. , Hong M. H. , Felip E. , Hall R. , Juan-Vidal O. , Brungs D. , Lu S. , Garassino M. , Chargualaf M. , Zhang Y. , Howarth P. , Uema D. , Lisberg A. , Sands J. , for the TROPION-Lung01 Trial Investigators , Martinengo G. L. , Puig J. , Brungs D. , Gao B. , Nagria A. , Karapetis C. , Parakh S. , Park J. , Catala G. , Forget F. , Ocak S. , Basappa N. , Liu G. , Menjak I. , Shieh B. , Lu S. , Luo F. , Sun H. , Wang J. , Yao Y. , Zemanova M. , Bennouna J. , Girard N. , Greillier L. , Lamour C. , Lena H. , Mascaux C. , Mazieres J. , Moro-Sibilot D. , Pérol M. , Pons-Tostivint E. , Westeel V. , Atmaca A. , Greil C. , Reinmuth N. , Schumann C. , Wehler T. , Wolf J. , Ho J. , Bocskei C. , Cappuzzo F. , de Marinis F. , Proto C. , Mencoboni M. , Novello S. , Salvagni S. , Parra H. S. , Daga H. , Goto Y. , Hayashi H. , Kitazono S. , Yoh K. , Ko R. , Kondo M. , Kozuki T. , Kurata T. , Mizutani H. , Ohashi K. , Oizumi S. , Okamoto I. , Sakaguchi S. , Ozasa H. , Sugawara S. , Tambo Y. , Tamiya M. , Tanaka H. , Okamoto I. , Lopez-Lopez F. , Ramirez Godinez F. J. , Rodriguez Cid J. R. , Cornelissen R. , Gans S. , Stigt J. , Kowalski D. , Lowczak A. , Milanowski J. , Mróz R. , Ramlau R. , Acosta-Rivera M. , Ahn M. J. , Chae Y. S. , Hong M. H. , Kang J. H. , Kim S. W. , Kim J. S. , Kim S. H. , Lee K. H. , Lee Y. G. , Shim B. Y. , Ledin E. , Poddubskaya E. , Daniel Chan B. Y. , Jain A. , Tan C. S. , Areses M. C. , Cobo M. , Domine M. , Felip E. , Pradera J. F. , Isla D. , Vidal O. J. , Majem M. , Paz-Ares L. , Provencio M. , Reguart N. , Baz D. V. , Cerciello F. , Früh M. , Chang W. C. , Chang G. C. , Huang W. T. , Ling C. C. , Wei Y. F. , Yang T. Y. , Ahmad T. , Gomes F. , Mansy T. , Bruno D. , Castine M. , Fang B. , Garassino M. , Hall R. , Halmos B. , Jahangir K. , Johnson M. , Johnson T. , Kalmadi S. , Lawler W. , Lisberg A. , Sadiq A. , Sands J. , Solomon B. , and Veatch A. , Datopotamab Deruxtecan Versus Docetaxel for Previously Treated Advanced or Metastatic Non-Small Cell Lung Cancer: The Randomized, Open-Label Phase III TROPION-Lung 01 Study, Journal of Clinical Oncology: official Journal of the American Society of Clinical Oncology. (2025) 43, no. 3, 260–272, 10.1200/jco-24-01544, 39250535. 39250535 PMC11771353

